# Effects of Rice Husk Biochar Coated Urea and Anaerobically Digested Rice Straw Compost on the Soil Fertility, and Cyclic Effect of Phosphorus

**DOI:** 10.3390/plants11010075

**Published:** 2021-12-27

**Authors:** Ashoka Gamage, Ben Basnayake, Janendra De Costa, Othmane Merah

**Affiliations:** 1 Department of Chemical and Process Engineering, Faculty of Engineering, University of Peradeniya, Peradeniya 20400, Sri Lanka; 2 Department of Agricultural Engineering, Faculty of Agriculture, University of Peradeniya, Peradeniya 20400, Sri Lanka; nri.srilanka@gmail.com; 3 Department of Crop Science, Faculty of Agriculture, University of Peradeniya, Peradeniya 20400, Sri Lanka; janedrad@gmail.com; 4 Laboratoire de Chimie Agro-Industrielle (LCA), Université de Toulouse, INRA, 31030 Toulouse, France; 5 Département Génie Biologique, Université Paul Sabatier, IUTA, 32000 Auch, France

**Keywords:** anaerobically digested compost, cyclic effect, leaching, rice husk biochar, slow-releasing fertilizer

## Abstract

Fertilizer application in rice farming is an essential requirement. Most of the high-yielding varieties which are extensively grown throughout the country require recommended levels of fertilizers to obtain their potential yields. However, effective, and efficient ways of fertilizer application are of utmost importance. Coated fertilizers are used to reduce leaching nutrients and improve the efficiency of fertilizer. However, conventional coated fertilizers such as Sulphur coated urea and urea super granules are not popular among rice farmers in Sri Lanka owing to the high cost. Mixing urea-coated rice husk biochar causes a slow release of nitrogen fertilizer. This coated fertilizer and rice straw compost reduction the cost of importations of nitrogen-based fertilizers per unit area of cultivation. The study aimed to evaluate the effects of rice husk biochar coated urea and anaerobically digested rice straw compost on the soil fertility, and the cyclic effect of phosphorus. Concerning the pot experiment, rice grain yield was significantly higher in Rice husk biochar coated urea, triple super phosphate (TSP), and muriate of potash (MOP) with anaerobically digested rice straw compost. The lowest yield was observed in the control. The release of phosphate shows a cycle effect which is an important finding. Rice husk biochar coated urea can potentially be used as a slow-releasing nitrogen fertilizer. In addition, the urea coated with biochar is less costly and contributes to mitigating pollution of water bodies by inorganic fertilizers (NPK).

## 1. Introduction

Agriculture plays an important and strategic role in the performance of the Sri Lankan national economy contributing nearly 8.4% of the gross domestic product (GDP) [1]. Presently, Sri Lanka produces 2.3 million metric tons (MMT) of rough rice (paddy) with a national average yield of 4.3 MT/ha in the 2017/2018 *Maha* seasons [1]. 

With the introduction of High Yielding Varieties (HYV), the provision of incentives to apply more chemical fertilizers. This was initiated in Sri Lanka in the year 1962. The HYVs are highly responsive to chemical fertilizers and hence it was essential to apply fertilizers to achieve expected yields. The increased pressure to maintain a high level of rice output for consumption has resulted in the increased use of inorganic fertilizer in rice fields in Sri Lanka [2]. The subsidy was limited to the main nitrogen fertilizer urea during 1997–2004. In the year 2005, the subsidy has again been expanded. Since 2005–2015, the subsidy provided for all three main fertilizers (N, P, and K) at a fixed price. 

At present, urea is the foremost nitrogen fertilizer (60–70%) used in Sri Lanka. Urea is the major source of supply of nitrogen fertilizer to crop production. Urea was imported to Sri Lanka during 2015–2017 has been for agricultural use. Urea has a nitrogen content of 46% is used in rice cultivation. The recovery of applied nitrogen to wetland rice is around 20–40% [3,4,5,6]. However, N application rates on agricultural fields often exceed the actual crop use and the unused N in the soil profile is either removed through leaching, denitrification, or volatilization. The nitrate ion is negatively charged and is not retained by the soils; thus, the dominant form of nitrate is leached from the soil [7,8]. Nitrate leaching which causes contamination of groundwater has become a major concern worldwide, mainly because of the intensification of agricultural productivity [9]. Rising nitrate concentration in groundwater has been detected in developing countries such as India and Sri Lanka where agricultural production has been intensified through the application of urea above the recommended levels [10]. Studies conducted on groundwater within intensively cultivated areas typically had nitrate-nitrogen concentrations in the range of 10–15 mg N/L compared to 0.2 mg N/L in non-cultivated lands. There is a correlation between groundwater quality and land use [11]. WHO standard in the year 2011 for nitrates concentrations in drinking water is 10 mg N/L. 

Many phosphorus (P) sources contaminate surface waters. These sources are agriculture, municipal sewage treatment plants, individual septic treatment systems, decaying plant material, runoff from urban areas and construction sites, stream bank erosion, and wildlife. The addition of phosphorus to surface water accelerates the eutrophication process, in which the water becomes overly enriched with nutrients [6]. Clune [12] indicated that total P should not exceed ranged from 0.010 to 0.053 mg /L in a stream and revers.

Biochar is a charcoal carbon product derived from biomass that enhances soils, sequesters or store carbon, and provides useable energy. Biochar is produced by thermal decomposition of organic material under an anaerobic condition (or limited supply of oxygen) and at relatively low temperatures (<700 °C) [13]. 

Biochar is a porous substance containing high levels of carbon and various functional groups. The addition of biochar to agricultural soil enhances soil physicochemical properties [14], enzymes activity, microbial activity, and microbial diversity [15]. However, the interactions between biochar and microbial activities in soil are not fully understood [15]. The application of biochar to soil could increase soil nutrient levels and crop productivity by reducing leaching losses and even supplying nutrients to the crops.

Mixing both urea and biochar in soils creates a slow release of nitrogen fertilizer and increases nitrogen retention [16], thus increasing the efficiency and reducing the cost of nitrogen-based fertilizers. Biochar has been found to adsorb a variety of heavy metals, including lead (Pb), arsenic (As), and cadmium (Cd) [17]. Biochar also slowed the downward movement of pesticides, thus potentially reducing the risk of groundwater contamination [18]. There are only a few methods available for rice husk and rice straw reuse in Sri Lanka. A significant amount of rice husk and straw remains unused and burned in open fields, causing serious environmental and health problems. The concept of producing biochar using rice husk and producing rice straw compost is practicable in Sri Lanka because about 1.8 million farm families are engaged in rice cultivation island wide. It is of utmost importance to manage the agricultural wastes while providing a value-added useful product for increasing agricultural productivity and protecting the environment. Therefore, this study aimed to evaluate the effects of rice husk biochar coated urea and anaerobically digested rice straw compost on soil fertility, and the cyclic effect of phosphorus. Furthermore, determine the behavior of rice husk biochar coated urea and evaluate the leaching losses of nutrients.

## 2. Results 

### 2.1. Proximate Analysis of Soil

The results indicated that the physicochemical properties of soil had much more influence on the morphological characters of the growth of rice plants (Table 1). Soil electrical conductivity indicates soluble nutrients, and it is useful in monitoring the mineralization of organic matter in -rich soil. The soil pH regulated the enzyme activity of soil and helped the solubility of the nutrients. Bulk density is an indicator of soil compaction, increases with time as particles settle after puddling is halted. Bulk density is inversely related to many important soil properties. These are water-holding capacity, soil particle size, total porosity, infiltration capacity, hydraulic conductivity, gas exchange, and nutrient mobility.

### 2.2. Pot Experiment

#### Rice Plant Vegetative Growth

**Figure 1:**
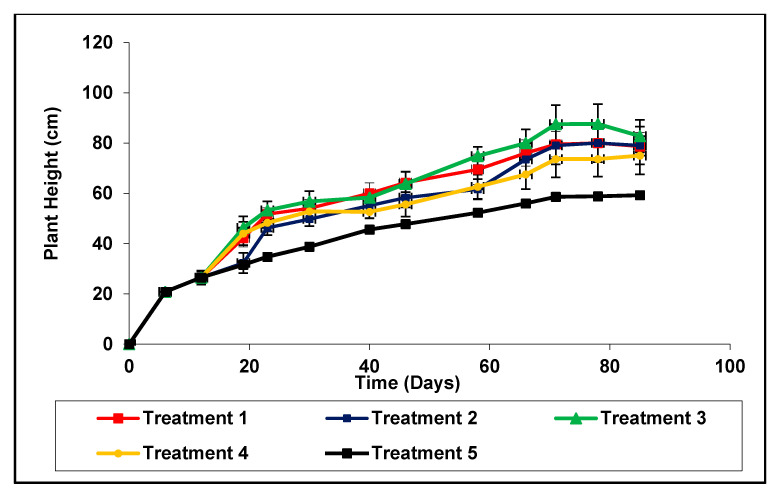
Variation of plant height with time in the pot experiment. Each data point is a mean of four replicates. Treatment 1− inorganic fertilizer only (Urea, TSP and MOP), Treatment 2− rice husk biochar coated Urea, TSP and MOP, Treatment 3− inorganic fertilizer (Urea, TSP and MOP) with anaerobically digested rice straw compost only, Treatment 4− rice husk biochar Urea, TSP and MOP with anaerobically digested rice straw compost and Treatment 5− no fertilizer as control.

The significantly highest (*p* < 0.05) plant height can be observed in the inorganic fertilizer followed by anaerobically digested rice straw compost treatment (treatment 3) while the control had the lowest. However, plant height was faster in the treatment using inorganic fertilizer (treatment 1) than in the biochar coated urea treatment (treatment 2) from 12 to 71 days after transplanting. After 71 days, plant heights were similar in all treatments (Figure 1). The number of tillers was highest in treatments 1, followed by 3 and 4 compared to the other treatments (Table 2). All fertilizer treatments, irrespective of the use of urea coated biochar or compost showed a significantly (*p* < 0.05) higher number of panicles per hole than the unfertilized control (Table 2). However, there were no significant differences in tiller number between different fertilizer combinations. Rice grain yield was significantly greater in treatments 4 and there was no significant difference in treatment, 2, 3, and 1 (Table 2). The lowest yield was observed in treatment 5 which is control and no addition of inorganic fertilizers.

There was a significant increase in biomass (dry matter) of the rice plant with the application of amendments as compared to the control (Table 2). The mean total dry biomass for treatment 4 was significantly higher than the means for all other treatments. The mean total dry biomass for treatments 1 and 3 was significantly higher than the means for treatments 2 and 5. The mean total dry biomass for treatment 2 (biochar coated urea) was significantly higher than that of treatment 5.

**Figure 2:**
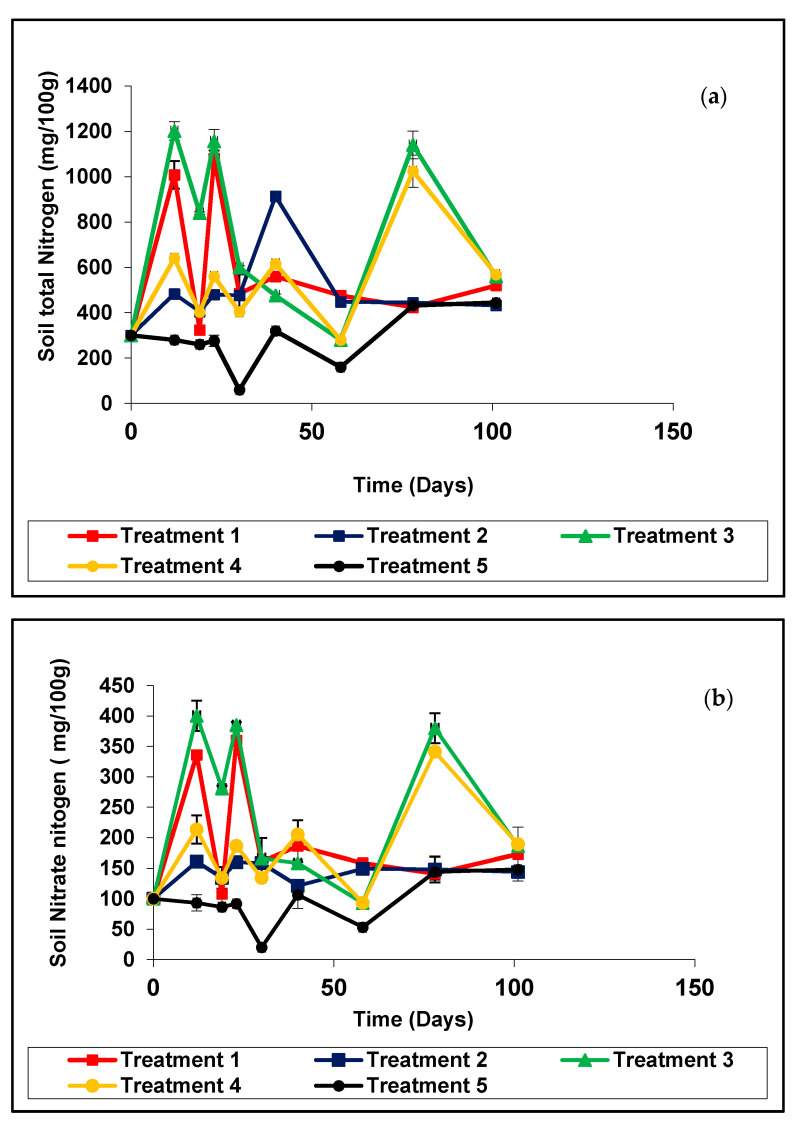
Variation of (**a**) total nitrogen in soil with different treatments; (**b**) nitrate nitrogen in soil with different treatments in the pot experiment.

Total N (organic and inorganic N) of the soil of treatment 1 increased during the first 12 days and then gradually decreased until 19 days and subsequently increased (Figure 2a). Total N in the soil of treatment 2 was maintained almost the same throughout the period. Total N in the soil of treatment 3 increased during the first 12 days and later on decreased gradually until days after planting and again increased. Soil nitrate-nitrogen of treatment 1 increased during the first 12 days and then gradually decreased until 19 days and subsequently increased (Figure 2b). Nitrate in the soil of treatment 2 was maintained almost the same throughout the period. 

Nitrate- N in the soil of treatment 3 increased during the first 12 days and later on decreased gradually until days after planting and again increased. According to the mean separation, treatments 3, 1, and 4 had the highest total N with no significant difference among them while 5 showed the lowest total N. The rice husk biochar coated urea and anaerobically digested rice straw compost amendment also caused a significant increase in the total plant N compared to control treatments. After three and half months, the soil N were as follows; treatment 1 (0.248 kg/ha), treatment 2 (0.207 kg/ha), treatment 3 (0.260 kg/ha), treatment 4 (0.247 kg/ha) and treatment 5 (0.112 kg/ha). After three and half months, the soil nitrate-nitrogen were as follows; treatment 1 (0.094 kg/ha), treatment 2 (0.069 kg/ha), treatment 3 (0.086 kg/ha), treatment 4 (0.082 kg/ha) and treatment 5 (0.037 kg/ha). 

**Figure 3:**
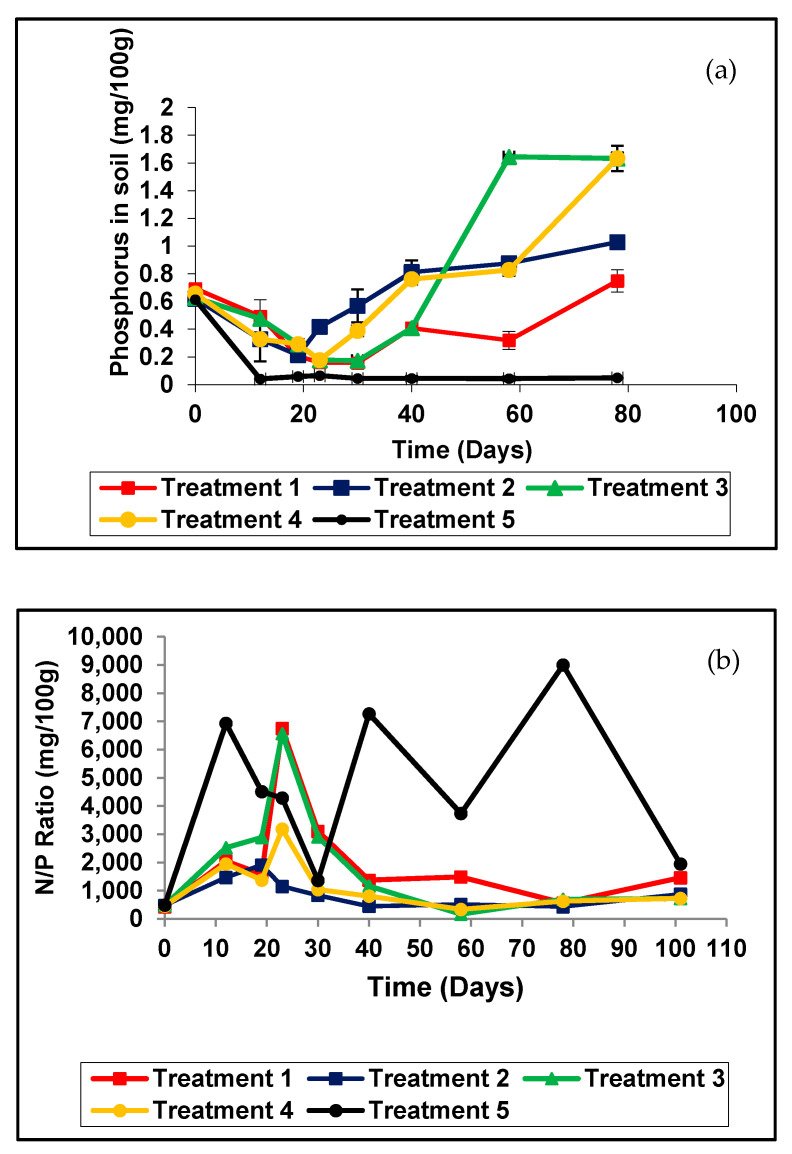
Phosphorus and N/P ratio variation. (**a**) Variation of phosphorus in soil with different treatments; (**b**). Influence of nitrogen on phosphorous uptake as illustrated with the ratio of nitrogen to phosphorus in the pot experiment. Each data point is a mean of four replicates. Treatment 1− inorganic fertilizer only (Urea, TSP and MOP), Treatment 2− rice husk biochar coated Urea, TSP and MOP, Treatment 3− inorganic fertilizer (Urea, TSP and MOP) with anaerobically digested rice straw compost only, Treatment 4− rice husk biochar Urea, TSP and MOP with anaerobically digested rice straw compost and Treatment 5− no fertilizer as control.

The P of the soil of treatment 1 decreased during the first 30 days and then gradually increased. P in the soil of treatment 2 decreased during the first 19 days and then gradually increased (Figure 3a). P in the soil of treatment 3 decreased during the first 30 days and then gradually increased. P in the soil of treatment 4 decreased during the first 23 days and then gradually increased. 

The rice husk coated urea (Treatment 2) and inorganic fertilizer with anaerobically digested rice straw compost amendment also caused a significant increase in the P compared to control treatments. After three and half months, the soil P were as follows; treatment 1 (0.356 kg/ha) treatment 2 (0.492 kg/ha), treatment 3 (0.780 kg/ha), treatment 4 (0.780 kg/ha) and treatment 5 (0.228 kg/ha). Phosphorus amounts in the soil of treatment 3 and 4 were the highest when compared to treatment 1. 

**Figure 4:**
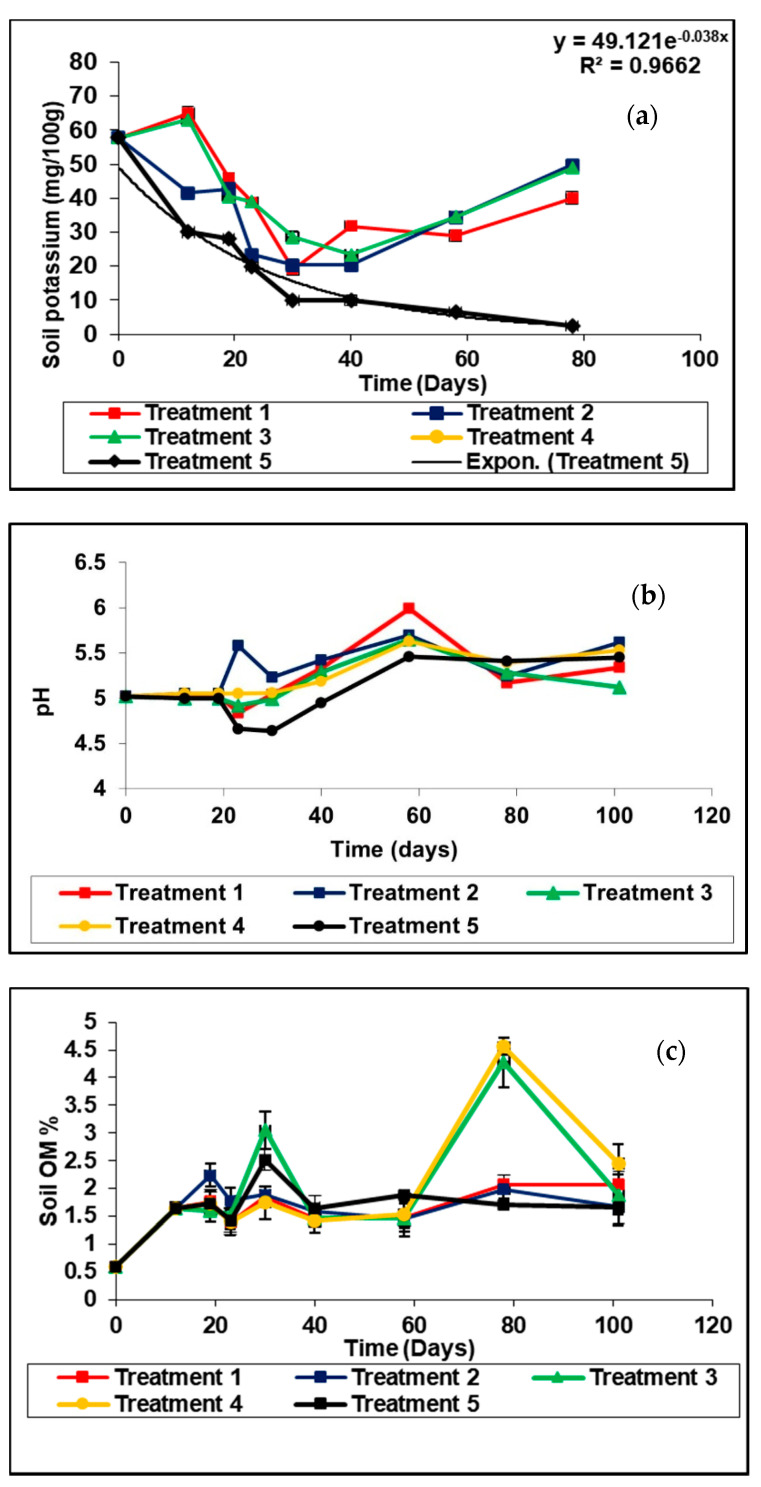
Variation of (**a**) potassium in soil with different treatments; (**b**) pH of the soil with different treatments; (**c**) organic matter of soil with different treatments in the pot experiment. Each data point is a mean of four replicates. Treatment 1− inorganic fertilizer only (Urea, TSP and MOP), Treatment 2− rice husk biochar coated Urea, TSP and MOP, Treatment 3− inorganic fertilizer (Urea, TSP and MOP) with anaerobically digested rice straw compost only, Treatment 4− rice husk biochar Urea, TSP and MOP with anaerobically digested rice straw compost and Treatment 5− no fertilizer as control.

After three and half months, the soil K was as follows; treatment 1 (23.52 kg/ha), treatment 2 (23.88 kg/ha), treatment 3 (24.72 kg/ha), treatment 4 (24.08 kg/ha) and treatment 5 (1.2 kg/ha). Considering the pH of the soil (Figure 4b) and organic matter content (Figure 4c), treatment effects were not significant (*p* < 0.05). 

### 2.3. Leaching Column Experiment 

Each data point represents the mean of two replicates. Treatment 1–inorganic fertilizer only, Treatment 2–rice husk biochar coated urea only, Treatment 3–anaerobically digested rice straw compost, and Treatment 4–no fertilizer as a control.

**Figure 5:**
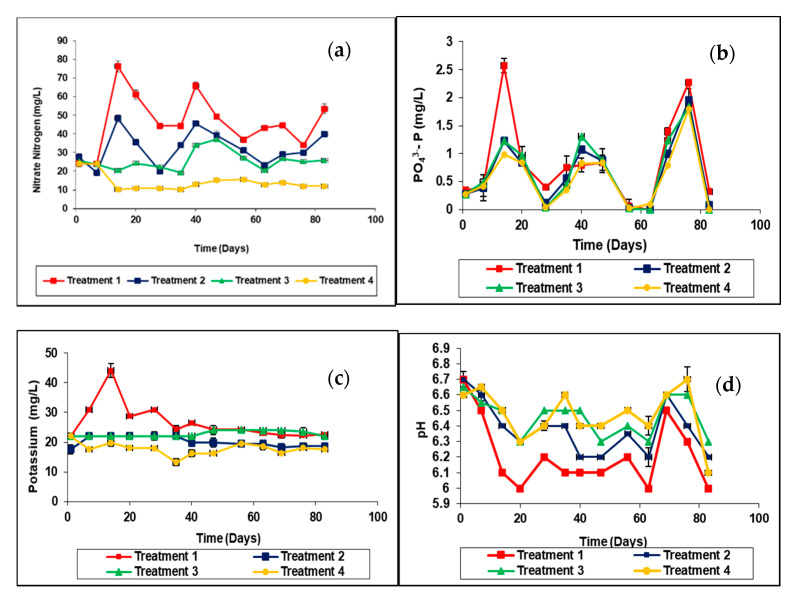
(**a**) Variation of NO_3_^−^-N with time; (**b**). Variation of PO_4_^−3^-P with time; (**c**). Variation of K+ with time; (**d**). Variation of pH with time in the leaching column experiment.

Figure 5a shows that the NO_3_-N of leachates of treatment 1 increased during the first 14 days and gradually decreased and again increased. The NO_3_-N of leachates of treatment 2 increased to 48.4 mg/ L during the first 14 days and decreased gradually and again increased. These fluctuations can be attributed to the application of urea at specified times responding to given dosages. In the case of compost, the NO_3_-N of leachates of treatment 3 increased during the first 47 days and then it decreased gradually 20.43 mg/L. In the first week, leaching losses from treatment 3 were higher than treatment 1.

According to the results of mean separation, the results of all the treatments are significantly different from each other (*p* < 0.0001). Treatment 1 has the highest value of NO_3_-N leaching and 2, 3, and 4 come in descending order (Figure 5a). According to Table 3 the cumulative NO_3_^−^-N leaching losses of kilogram per hectare (soil depth was 20 cm). of different treatments were as follows; treatment 1 was 30.1 ± 0.31 kg/ha, treatment 2 was 21.64 ± 0.51 kg/ha, treatment 3 was 16.64 ± 0.21 kg/ha and treatment 4 control were 9.26 ± 0.11 kg/ha. Treatment 1 has the highest value of NO_3_^−^-N leaching and 2, 3, and 4 come in descending order. Results reveal that biochar coated urea and rice compost have potential in terms of reducing groundwater pollution of NO_3_^−^ due to leaching compared to treatment 1 (inorganic fertilizer only).

The cumulative leaching losses of P of eighty-three days of different treatments were as follows; treatment 1 was 10.98 mg/L (0.66%), treatment 2 was 8.39 mg/L (0.19%), treatment 3 was 8.7 mg/L (0.14%) and treatment 4, control was 7.2 mg/L. According to the cumulative addition of phosphorous to the columns except for the control, leaching losses of P percentages are as follows; treatment 1 was 0.66%, treatment 2 was 0.19% and treatment 3 was 0.14%. Considering, P there are significant treatment effects (Figure 5b). According to Table 3 the cumulative P leaching losses in different treatments were as follows; treatment 1 was 0.544 kg/ha, treatment 2 was 0.419 kg/ha, treatment 3 was 0.435 kg/ha and treatment 4 control was 0.360 kg/ha. So, treatment 1 has the highest value of P leaching, and 2, 3, and 4 come in descending order. Using biochar as a coating material of urea also can save the reduction loss of P biochar coated urea and rice straw compost has potential in terms of reducing groundwater pollution of P due to leaching compared to treatment 1.

The cumulative leaching losses of K after eighty-three days of different treatments were as follows: treatment 1 (346.64 mg/L), treatment 2 (262.36 mg/L), treatment 3 (296.48 mg/L), and treatment 4 control (231.94 mg/L). According to the cumulative addition of potassium to the columns except for the control, leaching losses of K percentages are as follows; treatment 1 was 19.26%, treatment 2 was 5.05% and treatment 3 was 17.78%. According to the mean separation treatment, 1 (4.375 kg/ha) showed the highest K followed by 3 (3.709 kg/ha), 2 (3.294 kg/ha), and 4 (2.915 kg/ha). However, treatments 3 and 2 are not significantly different and again 2 and 4 are also not significantly different (Figure 5c). Treatments 4 and 3 have a higher pH than treatments 1 and 2 (Figure 5d).

## 3. Discussion

### 3.1. Analysis of Soil Physicochemical Properties 

Soil physicochemical properties changes caused by cultural practices and their consequences to soil productivity have generated significant research concerns. Degradation of soil fertility and quality are key factors for declining yield [19]. Soil fertility must be maintained to sustain and improve crop growth and yield. Soil is puddled (mixes soil and water to produce an impervious layer) before rice transplanting and kept flooded to create anaerobic conditions for rice growth. After puddling create a plow layer. This plow layer reduces hydraulic conductivity to support water ponding. It minimized the water percolation losses and enhanced the water and nutrient use efficiency of rice [20]. Furthermore, puddling triggered several changes in soil physical properties such as breaking down soil aggregates and forming hardpans at a shallow depth. This hard fan led to induced changes in pore size distribution [21]. 

Under flooded conditions, the redox potential of paddy is low, and NO_3_^−^, Fe^3+^, Mn_4_^+^, and SO_4_^2−^ are, respectively, reduced to NH_4_^+^, Fe^2+^, Mn^2+^, and S^2−^. Thus, flooding also improves the availabilities of P, K, Si, Mo, Cu, and Co and reduces the availabilities of N, S, and Zn. the redox potential is increased, thereby oxidizing the soil nutrient elements, and changing the effectiveness of the above-mentioned elements [22]. In most lowland rice soils, P availability initially increased on flooding and rice may meet its P requirement from the residual P [23]. The efficiency of K fertilizer application is affected by various factors. Both rice and the subsequent crop remove enormous amounts of K as a byproduct, resulting in a significant negative K balance in soils regardless of whether K fertilizers are applied at recommended doses [24].

### 3.2. Pot Experiment

#### 3.2.1. Rice Plant Vegetative Growth

The vegetative growth stage of rice has several phases. These phases are tillering, an increase in plant height, and leaf emergence at regular intervals. The length of this stage primarily determines the total life cycle duration of different rice varieties. Panicle initiation (PI) is the start of the reproductive phase of rice development that may occur before the maximum tiller number is reached in very-short duration and some short-duration varieties. Heading, when the panicle is fully visible stage rice varieties may be staggered due to later tillers that produce panicles. The maximum tiller (tillers develop in the axils of leaves, giving each plant three to a dozen or more) number is reached and followed by a vegetative lag phase before panicle initiation (PI) occurs [25]. In direct-seeded rice fields with a normal plant population (10 to 20 plants per square foot), rice plants generally produce 2 to 5 panicle bearing tillers per plant compared to 10 to 30 tillers per plant in transplanted rice where more space is available between plants. After maximum tillering has occurred, no more effective tillers are produced. A portion of the late tillers generally dies due to competition effects. The first yield component, potential panicles per unit area, is determined at this time [25]. Generally, the number of tillers is determined during the vegetative growth period and is mainly governed by the tillering capacity of cultivars, planting density, and the availability of mineral nutrition, particularly nitrogen [26]. Management of N fertilizer for maximum uptake efficiency by the rice crop varies with the cultural system, variety, soil texture, soil moisture, and several other factors. Correct management of the pre-flood N is critical since a rice crop’s potential grain yield is determined by the early N. The number of panicles (heads) and the number of grains per panicle are determined by the pre-flood N application [25,27,28]. These results indicated that the importance of basal application of readily available inorganic fertilizers especially N fertilizers.

#### 3.2.2. Yield and Yield Component

The reproductive stage is characterized by culm elongation, a rate of increase in tiller number, booting, the emergence of the flag leaf, heading, and flowering. The reproductive stage usually lasts approximately 60 days in most varieties. Panicle initiation is the time when the panicle primordia initiate the production of a panicle in the uppermost node of the culm. Panicle differentiation is closely associated with the internodal elongation stage. Panicle differentiation is equivalent at this point, the panicle is 1 to 2 mm in length and the branching of the panicle is visible. This is a critical stage during rice plant development and the environment can have a major effect on plant growth [25]. High temperatures tend to reduce the grain filling period and may reduce grain weight. Low temperatures tend to lengthen the time required for grain fill and ripening [25]. 

#### 3.2.3. Biomass Yield 

There is some evidence that biochar can reduce the run-off of agricultural inputs such as nitrates as well as suppress N_2_O and CH_4_ emission from the soil to the atmosphere [29,30,31,32]. In this way, biochar may act to improve the efficiency of the use of nitrogen in the soil. It is important to note that the properties of biochar in soils are dynamic due to the Physicochemical and biological changes which occur over time [29,30,31,32]. The addition of biochar to agricultural soil enhances soil properties such as water retention capacity, soil quality, soil organic matter stability, and nutrient retention capacity, maintaining soil acidity level, organic carbon sequestration, greenhouse gases emission reduction, and microbiological activity [14,15].

#### 3.2.4. Total Nitrogen and Nitrate Nitrogen in the Soil

Most of the organic amendments supply low amounts of available N due to immobilization after organic matter decomposition and N mineralization. In addition, the N from organic matter is also involved in other soil processes such as nitrification and de-nitrification. The higher N uptake percentage in the treatment 3 (0.20%) followed by 1 (0.19%), 4 (0.19%), and 2 (0.179%) compared to the control (0.128%) can be attributed to higher amounts of available N in the soil. Moreover, the addition of inorganic fertilizer with composted rice straw could facilitate decomposition due to relatively low lignin content. Due to its low N content, rice straw has a high C/N which could induce N immobilization during decomposition in soil [28]. Biochar added plots receiving nutrients (NPK) sustained higher crop yield compared to control plots where yield declined rapidly. Results from semi-arid soils in Australia have shown a positive response to biochar in combination with fertilizer in pot trials [29]. A key consideration highlighted in several studies is the potential for biochar to immobilize previously plant-available N. This could be from the mineralization of labile, high C/N ratio of biochar drawing N into microbial biomass, sorption of ammonium, or sequestration of soil solution into fine pores [30].

#### 3.2.5. Phosphorus in Soil

Soil solution P concentrations are relatively low, the quantity of P in the soil solution at a given time is generally on the order of <1 kg/ha, or 1% of the total quantity of P in the soil [33]. Inorganic P occurs in soil, mostly in insoluble mineral complexes, some of them appearing after the frequent application of inorganic fertilizers. These insoluble, precipitated forms cannot be absorbed by plants [34]. Organic matter is also an important reservoir of immobilized P that accounts for 20–80% of P in soils [34]. Only 0.1% of the total P exists in a soluble form available for plant uptake [35] because of its fixation into an unavailable form due to P fixation. The addition of P to the soil as soluble commercial fertilizer or in the by-products causes an immediate increase in the P concentration of the soil solution (Figure 3a). This P then participates primarily in adsorption and precipitation processes, leading to fluctuating P values in the soil. Initially, these sorption processes are easily reversible and added P is readily available for plant uptake, susceptible to losses in runoff or leaching [33]. In the present pot experiment, there was no leaching or surface runoff. Overuse of N has not only caused low N use efficiency and soil acidification [36] but also resulted in a deleterious effect on rice quality, especially the appearance [37]. Figure 3b illustrates the influence of the nitrogen to phosphorous ratio in soil indicates higher uptake of available nutrients. In all the four treatments, where nitrogen has been applied, the ratios showed a decreasing trend towards the end of the experiment. As reported by [38], it implies that freely available or excess nitrogen promotes the uptake of phosphorous. Although biochar in Treatment 2 had much less nitrogen, it was available to the rice plants with moderate levels of P, in comparison to Treatments 3 and 4, thus resulting in the lowest ratio. On the other hand, Treatment 4, performed similarly to Treatment 2 because of biochar, making nitrogen readily available with the added advantage of rice straw compost that would have supplied phosphorus, resulting in much higher yields. Note the variations of the control without inadequate P compared to nitrogen in the soil. 

#### 3.2.6. Potassium in Soil

Potassium (K) is a macronutrient taken up by plants in large quantities. Potassium performs important roles such as enzyme activation, photosynthesis, photosynthate translocation, protein synthesis (i.e., nitrogen use), and plant water relations [6]. The potassium of soil of treatments 3 and 4 was the highest as compared to treatment 1, which contains NPK only. It could be attributed to the addition of compost at the beginning and during the growing period of the crop. A negative exponential function of the form $K=K_{0}e^{-\propto t}$ could be fitted to the variation of soil K in treatment 5. (Figure 4a), where K and Ko are potassium concentration at time t and initial concentration (57.88 mg/100g), respectively and α (0.47) is the adsorption coefficient of potassium in the soil. The trend line can be observed as shown in Figure 5 simulation. In all other treatments, precipitations of K have taken place with desorption coefficients of exponential functions soon after 40 days had lapsed. This result implies that the K levels remain high due to compost applications and bio-char additions even for the following season. A lower level of K concerning those treatments was found to be the case for the inorganic conventional system in Treatment 1, necessitating K applications for the next cropping season.

Because in the present pot experiment there was no leaching or surface runoff, when incorporated into soil substrate in pot trials, biochar, and local organic fertilizers altered the soil physical structure (bulk density) and modified the soil chemical properties (pH, CEC, and N, P and K supply). Low pH can limit plant growth by modifying the dynamic of crop nutrients. Hence, biochar addition could be particularly beneficial in acidic soils [39].

#### 3.2.7. Soil pH and Soil Organic Matter

The addition of organic matter to flooded soil raises the NH4^+^ concentration of floodwater and leads to a pH increase [40]. Decreasing soil pH following organic materials can be partially attributed to the high release of organic acids causing mobilization of native calcium present CaCO_3_ in the soil. According to Agusalim et al. [41], organic C content in rice husk biochar is lower than that of rice straw. The highest level of soil organic matter was observed in rice husk biochar treated soil. This phenomenon indicated the recalcitrant nature of carbon in rice husk biochar as has been suggested by many researchers [42,43].

### 3.3. Leaching Column Experiment 

#### 3.3.1. NO_3_^−^-N in the Leachate

Organic carbon decomposition by microbial activity influences many processes of the nitrogen cycle. With time, nitrogen concentration decreased due to microbial utilization of nitrate compounds and denitrifying as ammonia gas [44]. Anaerobically digested rice straw compost had readily available NO_3_^−^-N. Management of urea-containing fertilizers requires a clear understanding of the reactions that urea undergoes when added to the soils. Urea is hydrolyzed to ammonia and carbon dioxide through the action of a soil urease enzyme. In most soils, urea hydrolysis occurs rapidly after urea-containing fertilizers are applied. Available N in organic sources is present in NH_4_+ or NO_3_^−^ form but in amide form in urea. Hydrolysis of urea takes 1–2 weeks [45]. The cumulative NO_3_^−^-N leaching losses of eighty-three days of different treatments were as follows; treatment 1 was 602.16 mg/L, treatment 2 was 423.28 mg/L, treatment 3 was 332.28 mg/L and treatment 4 control was 185.24 mg/L. According to the cumulative addition of nitrogen to the columns except for the control, leaching loss of NO_3_^−^-N percentages is as follows; treatment 1 was 65.7%, treatment 2 was 41.2% and treatment 3 was 21.8%. The efficiency of the urea-N in rice culture is very low, generally around 30–40%, in some cases even lower [46]. The application of urea was 110 kg/ha. Concerning the treatment 1 inorganic fertilizer, the percentage NO_3_^−^-N leaching losses was 26.07%. One-fourth of the N is in the nitrate form and is subject to loss by leaching or denitrification [47]. The application of biochar to soil could increase soil fertility and crop productivity by reducing leaching. However, the effects of biochar on nutrient leaching and organic carbon retention have been reported to vary with the applied biochar pyrolysis temperature, raw material, and soil type [15].

#### 3.3.2. P in the Leachate

Microorganisms also play a central role in the natural phosphorus cycle. The use of phosphate solubilizing microorganisms plays a vital role in solubilizing the insoluble forms of phosphorus. Soil microorganisms act as sink and source of phosphorus (P) and mediate key processes in the soil P cycle, e.g., P mineralization and immobilization [48]. The fluctuations of phosphate may have cyclic effects which were 28 days (Figure 5b). The rate increased gradually to a peak and then reduced to lower values. It can be mathematically expressed as logistic growth equations, applicable for microbial growth and decay. The microbial cyclic effects have been recorded by [49,50]. This cycle occurs utilizing the cyclic oxidation and reduction of phosphorus compounds, where electron transfer reactions between oxidation stages range from phosphine to phosphate. The genetic and biochemical mechanisms of these transformations are not yet completely understood [51].

#### 3.3.3. K in the Leachate

According to Table 3, the cumulative K leaching losses of kilogram per hectare of different treatments were as follows; treatment 1 was 17.33 kg/ha, treatment 2 was 13.18 kg/ha, treatment 3 was 14.82 kg/ha and treatment 4 control was 11.59 kg/ha. Treatment 1 has the highest value of K leaching and 2, 3, and 4 come in descending order. Using biochar as a coating material of urea also saves the reduction loss of K. Biochar coated urea and rice straw compost have potential in terms of reducing groundwater pollution of K due to leaching compared to treatment 1.

#### 3.3.4. pH

There are also relationships between fertilizer application and NO_3_^−^-N and PO_4_^3−^-P leaching and pH. The maximum uptake of most nutrients occurs at a soil pH near neutral. The availability of most macronutrients such as nitrogen, phosphorus, potassium, sulfur, calcium, and magnesium, decreases as soil acidity increases. The relationship between soil pH and nutrient uptake efficiency is that fertilizer use, and crop response are expected to change as a function of soil pH [52]. 

## 4. Materials and Methods

### 4.1. Preparation of Slow-Releasing Nitrogen Fertilizer

**Figure 6:**
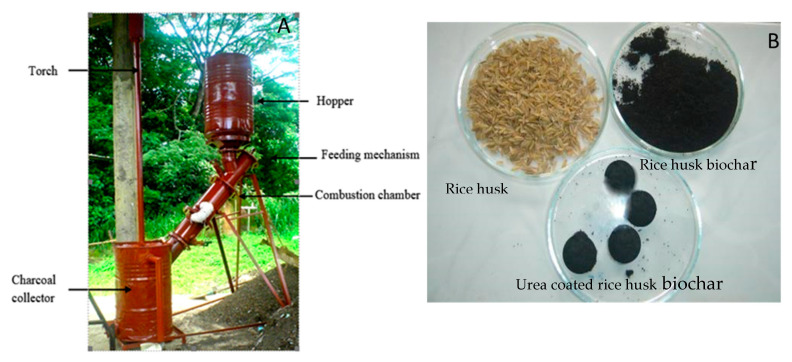
The material used for the biochar production and urea coated biochar generated. A. Pyrolizer; B. Slow releasing nitrogen fertilizer prepared using biochar.

Biochar was produced from rice husk [53]. The pyrolizer was designed to produce biochar. The pyrolizer consists of the following main components; i. Hopper for feedstock ii. Feeding mechanism iii. Combustion chamber (hollow cylindrical retort) iv. Charcoal collector v. Torch. The reactor was loaded with 160 kg rice husk. Then by using the feeder, a filled column of rice husk (20 kg) was obtained. Coconut husk and shells which were filled into the charcoal collector was used as a fuel source. It was fired from the charcoal remover. Once ignition had taken place, an airstream was introduced using a centrifugal blower. The quantity of air was regulated by the inlet valve. The torch was used, and an airstream was introduced into the inclined cylindrical chamber through the air inlet. Pyrolysis of rice husk takes place in the inclined chamber (Temperature, 550 °C), and hence, it is performed as an inclined flow reactor (Figure 6). The average temperature depends on the air mixing ratio of the incline chamber. A temperature measuring pyrometer probe was inserted into the reactor chamber and got the temperature. In this method, the quantity of feedstock, time taken to produce charcoal were measured and recorded. After cooling the reactor to room temperature, the char was collected from the reactor.

Rice husk was partially burnt, ground, sifted through sieve no 60 (250 µm) and obtained a fine powder. This fine powder was used as a coating material for urea. Carbon powder and urea were mixed thoroughly and with the help of cassava starch. Granules of 1.0 cm diameter were formed manually. Production of 1 kg of slow-releasing fertilizer mixture having a ratio of biochar: urea (2:1). As a binding agent 30–50 g of dried cassava starch was used (cassava boiled dried ground). Sodium hydroxide can be added about 3% of the weight of starch to enhance the strength and binding quality. After making carbon-coated urea balls, they were kept in a desiccator for about three days. The nitrogen content of urea was 46% and 0.3% in rice husk biochar (Figure 6).

### 4.2. Experimental Site for the Pot Experiment

A pot experiment was conducted in the *Yala* season at the Meewathura Farm, Department of Agricultural Engineering, Faculty of Agriculture, University of Peradeniya, Sri Lanka. Soil samples were collected from a farmer’s field at Megoda Kalugamuwa, Sri Lanka at depths of up to 20 cm, and then dried, ground, and passed through a 2.0 mm sieve. The soil samples were analyzed for total nitrogen, phosphorus, potassium, and soil samples were analyzed for organic matter, pH, EC, and Eh [54]. 

### 4.3. Pot Experiment

Soil samples were collected from a farmer’s field at Megoda Kalugamuwa, Sri Lanka at depths of up to 20 cm, and then dried, ground, and passed through a 2.0 mm sieve. Fifteen kg of soil was placed each into twenty plastic pots (size of the pot 30 cm diameter and 45 cm height) and the pots were tapped several times to settle the soil. Soils were saturated with water and allowed for one week to reach equilibrium. Rice seedlings (Bg 358) grown on Petri dishes for one week were planted in each pot (three plants per pot). The treatments were set up in a completely randomized design: Five treatments were used for twenty pots each replicated four times (Table 4).

Before the fertilizers were applied, the water level in each pot was maintained at 2 cm from the soil surface to ensure that the system was waterlogged. The water level was marked on the pots. The water level in the pots was maintained (for the vegetative growth period) by adding water as the deficit of the original water level. The fertilizers were applied to the soil surface. Urea (46 g N/100g), TSP (20 g P/100 g), and MOP (60 g K/100 g) were applied to all pots, except the control based on the Department of Agriculture (2009) recommendation. Rice husk biochar contains 0.58 g N/100 g, 0.12 g P/100 g, and 0.2 g K/100 g while rice straw compost 0.98 g N/100 g, 0.6 g P/100 g and 1.32 g K/100 g respectively. In the case of treatments 2 and 4, one-third of urea from the recommended amount (Urea: biochar- 1:2) per pot was applied. It was assumed that in treatments 2 and 4, rice husk biochar would help to reduce leaching losses of urea. Zinc Sulphate at the rate of 3.92 mg per pot was applied. Rice straw compost was applied at the rate of 2 tons/ha to treatments 3 and 4 twice during the cropping season. Compost additions to soil have the potential to contribute to the improvement of soil physical properties and fertility of the soil. 

### 4.4. Soil Characterization and the Measurement of Agronomic Parameters of Rice Plants

Two undisturbed soil samples of about 50 g were taken for bulk density and soil water determination, and a disturbing sample of about 100 g was taken for analysis of chemical properties. The soil samples were collected once a week after the application of basal dressing over three months. Soil texture, pH, EC, Eh, organic carbon, available P, total N, and exchangeable K were determined using standard soil analytical methods [54]. Herbicides or insecticides were not added to the treatments. Weed control was done manually. The number of tillers per hole was counted at the maximum stage of forming tillers (45 days after planting). Plant heights were measured once a week. Before harvesting, the number of panicles per hill was counted. At the time of harvesting 10 randomly selected rice plants were uprooted from each plot and roots were thoroughly washed and kept inside an oven at 70 °C for 3 days for dry weight determination of both below and above-ground biomass. At maturity, plots were harvested discarding the border rows and the grain yield of each plot was recorded at 14% moisture content. Three months after planting, plants were uprooted, and soils attached to roots were removed using running water and plants were oven-dried at 80 °C for 72 h and dry weight was recorded. 

### 4.5. Leaching Column Experiment

**Figure 7:**
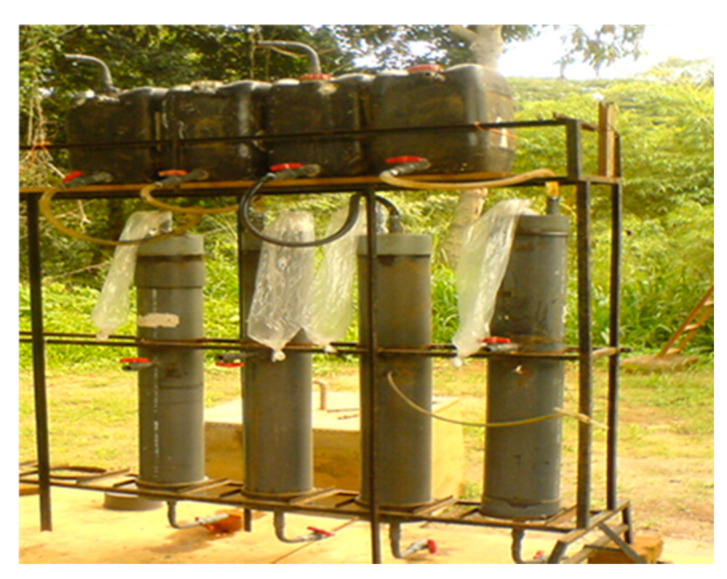
Setup of leaching columns.

Two sets of leaching columns were prepared using 1.2 m tall polyvinyl chloride (PVC) pipes with a diameter of 0.15 m (Figure 7). Gas measuring and leachate recirculation orifices were made at the top of the column. The gas collection was done by using polythene tubes. A sampling port was attached to the bottom end of the column cap, and it was closed until getting the samples.

There was a plastic container connected to the system to maintain a constant height of water (5 cm). Four PVC pipes of 1.2 m high and 0.15 m diameter were used to make leaching columns. A 40 mm thick layer of pre-cleaned, washed, and dried gravel with a particle size of 5–7.5 cm was used as the filter medium. Rice soil was taken from the Megoda Kalugamuwa, Kandy, Sri Lanka.

Every column was filled with 15 kg of rice soils (bulk density of 1.3 kg/m^3^) as a substrate and the columns were saturated with water for 2 weeks. Table 5 Shows the four treatments were used for the columns as follows:

According to the Department of Agriculture (2009) recommendations, fertilizer for 3 1/2 months variety was used for the columns except for the control. The Department of Agriculture (2009) recommendation was, 110 kg/ha of urea- (Basal dressing 10 and top dressing, 30, 50, and 20 kg/ha after 3, 5, 7, and 8 weeks after planting, respectively). 50 kg/ha of TSP, for basal dressing only, 80 kg/ha of MOP- basal, and 8 weeks after planting (30 and 30 kg/ha, respectively). ZnSO4, 5 kg/ha. Same amounts of N, P, and K were applied for each column surface area. The 100 ml leachates were obtained (once a week for 12 weeks period) from the leaching columns. Nitrate Nitrogen, phosphate phosphorus, potassium, and pH were determined using standard soil analytical methods [20]. A completely randomized design was used for this experiment. 

### 4.6. Statistical Analyses

Analysis of variance (ANOVA) was performed and differences in mean values were determined using *t*-test at *p* < 0.0001 and employing ANOVA and least significance difference procedures (SAS Institute (2011) SAS/STAT User’s Guide (Version 9.2). Statistical Analysis System Inst, Cary, NC, USA).

## 5. Conclusions

Concerning the pot experiment, rice grain yield was significantly higher in rice husk biochar coated urea, TSP, and MOP with anaerobically rice straw compost. (Treatment 4) applied to the soil. The lowest yield was observed in the control (treatment 5. Considering the pH of the soil and organic matter content of the pot experiment, treatment effects were not significant. The cyclic effect of phosphate release is an important finding, and it could be the central issue in defining microbial behavior in soils. The change of phosphate may have 28-day cyclic effects. Biochar can be used as a soil amendment and organic fertilizer, but adjustment of pH was required at high application rates. Rice husk biochar coated urea can potentially be used as a slow-releasing nitrogen fertilizer which reduces leaching of urea. Furthermore, the urea coating with biochar is less costly and helps to reduce the fertilizer cost (70% of urea cost) and contribute to mitigating pollution of water bodies. The addition of biochar to the soil significantly increased the soil properties such as pH, electrical conductivity, organic matter, available phosphorus, available potassium, and the C/N ratio. Biochar could act as a nutrient source and release nutrients such as N, P, and K. Rice straw and rice husks are rich in silicon. Carbonized straw or husk is used as a good silicon fertilizer. Silicic acid from the carbonized material, dissolved in the soil solution and can be adsorbed to soil minerals. Biochar application with the compost should be considered as an environmental and efficient agricultural practice for sustainable soil management in the agricultural ecosystem.

## Figures and Tables

**Table 1:** Physicochemical proprieties of the soil.

Properties	Soil
Moisture content %	2.94 ± 0.120 (air dried)
Bulk density (g/cm^3^)	1.3 ± 0.040
pH	5.2 ± 0.028
Carbon content %	0.6 ± 0.006
Total Nitrogen (mg/kg)	300 ± 8.16
Phosphorus (mg/kg	7 ±0.0.12
Potassium (mg/kg)	500 ± 10.8
Electrical conductivity (µS/cm)	89.1 ± 0.817
Salinity %	NA
Texture	Sandy loam

NA-not available. Each value represents the mean of three samples.

**Table 2:** Effect of different treatments on the growth and yield parameters of rice cultivated in pots.

Treatment	Dry Matter (g)	Number of Tillers per Pot	Number of Panicles per Pot	Yield (kg/ha)
1	63.02 ^b^ ± (0.93)	12.25 ^a^ ± (2.50)	8.0 ^a^ ± (0.50)	4359.18 ^b^ ± (948.00)
2	50.83 ^c^ ± (0.86)	9.75 ^ab^ ± (2.06)	7.66 ^a^ ± (0.90)	3763.26 ^b^ ± (771.00)
3	61.01 ^b^ ± (0.70)	11.25 ^a^ ± (0.50)	8.33 ^a^ ± (0.66)	4648.97 ^b^ ± (191.00)
4	65.03 ^a^ ± (1.25)	11.25 ^a^ ± (2.50)	8.10 ^a^ ± (0.52)	5081.63 ^a^ ± (115.10)
5	27.82 ^d^ ± (2.26)	7.25 ^b^ ± (0.95)	5.00 ^b^ ± (0.77)	891.83 ^c^ ± (163.36)

Mean followed by the same letter at each column are not significantly different (*p* < 0.05), each value represents the mean of four replicates. SD is given in parenthesis. Treatment 1− inorganic fertilizer only (Urea, TSP, and MOP); Treatment 2− rice husk biochar coated urea TSP and MOP; Treatment 3− inorganic fertilizer (Urea, TSP, and MOP) with anaerobically digested rice straw compost only; Treatment 4− rice husk biochar urea, TSP and MOP with anaerobically digested rice straw compost and Treatment 5− no fertilizer as the control.

**Table 3:** Leaching losses of the nutrients.

Nutrient	Treatment 1	Treatment 2	Treatment 3	Treatment 4
NO_3_-N (kg/ha)	30.10 ^a^ ± (0.31)	21.64 ^b^ ± (0.51)	16.64 ^c^ ± (0.21)	9.26 ^d^ ± (0.11)
PO_4_-P (kg/ha)	0.549 ^a^ ± (0.21)	0.419 ^b^± (0.45)	0.435 ^b^ ± (0.16)	0.360 ^b^ ± (0.39)
K (kg/ha)	4.357 ^a^ ± (0.95)	3.294 ^b^± (0.32)	3.709b ^c^ ± (0.21)	2.915 ^c^ ± (0.98)
Gas volume (m^3^/ha)	15,952.74 ^a^ ± (12.31)	8966.34 ^b^ ± (15.64)	8824.92 ^b^ ± (11.25)	7467.24 ^b^ ± (14.56)

Mean followed by the same letter at each column are not significantly different (*p* < 0.05), each value represents the mean of two replicates. SD is given in parenthesis.

**Table 4:** Treatments were carried out in this study.

Treatment Number	Fertilizer Mixture
1	Inorganic fertilizer only (Urea, TSP, and MOP)
2	Rice husk biochar coated urea, TSP, and MOP
3	Inorganic fertilizer (Urea, TSP, and MOP) with anaerobically digested rice straw compost [55,56] only
4	Rice husk biochar coated urea, TSP, and MOP with anaerobically digested rice straw compost anaerobic digestion [55,56]
5	No fertilizer added

**Table 5:** Treatments were carried out in this study.

Treatment Number	Fertilizer Mixture
1	Inorganic fertilizer only (Urea, TSP, and MOP)
2	Rice husk biochar coated urea, TSP and MOP only
3	Anaerobically digested rice straw compost [55,56]
4	No fertilizer as a control

## Data Availability

Not applicable.
